# What Is Best for Weight Loss? A Comparative Review of the Safety and Efficacy of Bariatric Surgery Versus Glucagon-Like Peptide-1 Analogue

**DOI:** 10.7759/cureus.46197

**Published:** 2023-09-29

**Authors:** Nimra Klair, Utkarsh Patel, Ayushi Saxena, Dhara Patel, Ismat E Ayesha, Neetha R Monson, Shivana Ramphall

**Affiliations:** 1 Internal Medicine, California Institute of Behavioral Neurosciences & Psychology, Fairfield, USA; 2 Medicine, California Institute of Behavioral Neurosciences & Psychology, Fairfield, USA

**Keywords:** glucagon-like peptide-1 receptor agonist, ozempic, overweight, obesity, weight loss, sleeve gastrectomy, bariatric surgery, semaglutide, wegovy, glp-1 agonist

## Abstract

Obesity is a global health concern, necessitating effective weight-loss interventions. This study aimed to compare the efficacy and safety of semaglutide, a pharmacotherapeutic option, with bariatric surgery, a commonly utilized surgical intervention, for weight reduction. A systematic review of clinical trials, including the STEP (Semaglutide Treatment Effect in People) trials, sustain trials, pioneer trials, and the STAMPEDE (Surgical Treatment and Medications Potentially Eradicate Diabetes Efficiently) trial, was conducted to evaluate the outcomes of these interventions. The analysis of the clinical trials revealed that semaglutide demonstrated significant weight reduction in participants. However, adverse effects such as gastrointestinal (GI) disturbances, increased pulse rate, and rare cases of thyroid cancer were observed. Long-term effects showed partial weight regain and a return of certain cardiometabolic variables to baseline levels after semaglutide withdrawal. Comparatively, bariatric surgery, as demonstrated in the Longitudinal Assessment of Bariatric Surgery (LABS) consortium and supported by the STAMPEDE trial, exhibited higher efficacy in weight reduction and the management of obesity-induced complications such as diabetes. The STAMPEDE trial demonstrated that bariatric surgery, specifically Roux-en-Y gastric bypass (RYGB) and sleeve gastrectomy (SG), led to a significantly higher percentage of patients achieving desired diabetes treatment targets compared to medical therapy alone. While bariatric surgery showed superior efficacy, it also carried a higher risk of complications. In contrast, semaglutide presented a noninvasive alternative with significant weight reduction and lower incidences of adverse effects. In conclusion, this study highlights that bariatric surgery, such as Roux-en-Y gastric bypass and sleeve gastrectomy, remains a highly effective intervention for weight loss and management of obesity-induced complications. However, semaglutide represents a valuable noninvasive alternative, offering significant weight reduction and lower risks of adverse effects. The choice between these interventions should be based on individual patient characteristics and a comprehensive assessment of the risk-benefit profile.

## Introduction and background

The prevalence of obesity worldwide, characterized by a body mass index (BMI) of 30 kg/m^2^ or higher, has experienced a substantial surge over the years. In 1975, a mere 5% of the global population fell into the category of obesity, whereas by 2014, this figure had more than doubled to 13% [1]. Furthermore, around 4% of individuals can be classified as severely obese, with a BMI between 30.0 kg/m2 and 34.9 kg/m2, while 1% are afflicted by morbid obesity, defined by a BMI exceeding 35.0 kg/m2 [1-2]. Considerable disparities in the prevalence of obesity exist among adults in the United States, with over one-third of the population affected influenced by factors such as race and socioeconomic status [3].

According to some studies, gastrointestinal surgery offers unparalleled efficacy and enduring weight-loss outcomes for individuals grappling with obesity [4]. Bariatric surgery, also known as metabolic surgery, is increasingly recognized as a highly effective intervention for obesity and its associated conditions. It enables patients to achieve significant and sustainable weight loss, leading to improved overall health. In the United States, the number of procedures has surged by 24% since 2011, reaching a total of over 190,000 in 2015 [5-6]. The objectives of bariatric surgery encompass enhancing quality of life, reducing the risk of co-morbidities, achieving remission and improvement in metabolic diseases, and facilitating weight loss. These factors have undergone extensive research and are clearly defined in the existing literature. Well-established metrics exist to define the extent of improvement and remission of co-morbidities associated with obesity [7]. To be eligible for bariatric surgery, individuals must meet the current guidelines set by the National Institutes of Health. These guidelines stipulate that patients should have a body mass index (BMI) of 40 kg/m2 or higher or a BMI of 35 kg/m2 or higher with high-risk co-morbid conditions such as severe diabetes mellitus, life-threatening cardiopulmonary issues, or obesity-related physical problems that significantly impact their lifestyle. Moreover, patients should have previously attempted and failed diet and exercise interventions. They should also demonstrate motivation, be well-informed about the procedure, and not suffer from significant psychological disorders [8]. Laparoscopic techniques have revolutionized contemporary bariatric procedures, enabling the utilization of various surgical options. These include Roux-en-Y gastric bypass (RYGB), sleeve gastrectomy (SG), adjustable gastric banding (AGB), and bilio-pancreatic diversion with duodenal switch (BPDDS) [9-10].

Semaglutide, an investigational human glucagon-like peptide-1 analogue (GLP-1 analogue), is being developed as a potential treatment for type-2 diabetes (T2D). It shares a similar structure to liraglutide and exhibits a high degree of structural similarity (94%) to native human GLP-1 [11]. The motivation behind the development of semaglutide, a once-weekly GLP-1 receptor agonist (GLP-1 RA), stemmed from the desire to enhance the metabolic effects within the realm of GLP-1 receptor agonists (GLP-1 RA). This was prompted by the observation that albiglutide, dulaglutide, and exenatide once weekly were comparatively less effective than liraglutide in terms of inducing weight loss. Therefore, the development of semaglutide aimed to further optimize the metabolic benefits associated with GLP-1 RA [12-14]. According to a study conducted by Blundell et al., following the consumption of a standardized breakfast, treatment with semaglutide resulted in a decrease in ad libitum energy intake during subsequent meals, leading to a reduction in overall energy intake [15]. Notably, this effect was not accompanied by any changes in resting metabolic rate. The observed decrease in energy intake can be attributed to the suppression of appetite rather than to feelings of nausea or food aversion. Semaglutide treatment also exhibited a noteworthy impact on appetite, as it reduced feelings of hunger and food cravings while also decreasing the preference for high-fat foods. Additionally, semaglutide treatment demonstrated improved control over eating habits and portion sizes during meals [15].

In recent years, there have been significant advancements in the field of weight loss interventions, specifically focusing on the efficacy of semaglutide treatment and bariatric surgery. This review aims to compare the effectiveness of these two treatment modalities in achieving weight loss outcomes and will acknowledge the practical limitations of using either of these approaches for long-term weight management. While both approaches have shown promise in promoting weight loss, it is crucial to consider factors such as accessibility, cost-effectiveness, sustainability, and potential adverse effects when evaluating their practicality as weight loss interventions. By critically examining the recent developments and limitations of semaglutide treatment and bariatric surgery, this review aims to provide a comprehensive understanding of their respective efficacies and practical implications for individuals seeking long-term weight management solutions.

## Review

Efficacy of bariatric surgery for weight loss

In the United States, the number of bariatric procedures performed annually was estimated to be around 252,000 as of 2018. Among primary bariatric procedures, approximately 61% were sleeve gastrectomy procedures, while 17% were RYGB procedures. The AGB and biliopancreatic diversion procedures each accounted for less than 2% of the total [16]. According to the 1991 National Institutes of Health guidelines, individuals with a BMI of at least 40 kg or a BMI of at least 35 kg accompanied by serious obesity-related comorbidities should be considered for bariatric surgery. Despite being from the late 20th century, these guidelines are still being used [8]. As mentioned earlier, bariatric surgery is effective in weight loss, resulting in improvements in obesity-related comorbidities such as type-2 diabetes mellitus (T2DM), hypertension, dyslipidemia, urinary incontinence, sleep apnea, osteoarthritis, and even cancer [16]. Bariatric surgery has emerged as a significant advancement in the field, particularly in its impact on patients with type-2 diabetes mellitus. Notably, a systematic review and meta-analysis of observational and randomized trials, which included 706 patients with type 2 diabetes and follow-up ranging from 12 to 36 months, confirmed the superiority of bariatric surgery. The analysis showed that bariatric surgery was associated with a higher remission rate of type-2 diabetes (odds ratio [OR], 14.1 [95% CI, 6.7-29.9]; P < 0.001), a higher rate of glycemic control (OR, 8.0 [95% CI, 4.2-15.2]; P < 0.001), and a lower glycosylated hemoglobin (HbA1c) level (mean difference, -1.4% [95% CI, -1.9% to -0.9%]; P < 0.001) compared to medical treatment [17].

Bariatric surgery has shown long-term effectiveness in sustaining weight loss. The mechanisms contributing to sustained weight loss include changes in appetite regulation, altered gut hormone secretion, and modifications in the gut microbiota. A study published in the New England Journal of Medicine in 2017 reported that five years after bariatric surgery, patients maintained an average weight loss of 25% of their initial body weight [18]. The STAMPEDE (Surgical Treatment and Medications Potentially Eradicate Diabetes Efficiently) trial, a randomized controlled study exploring the effectiveness of metabolic surgery in individuals with diabetes, revealed compelling results. The trial included 150 patients with T2DM and a BMI ranging from 27 to 43 kg/m2. Participants were randomly assigned to one of three groups: Roux-en-Y Gastric Bypass (RYGB), Sleeve Gastrectomy (SG), or intensive medical therapy alone [19]. After one year, the study found that only 12% of patients in the medical therapy group achieved the desired diabetes treatment targets (HbA1c ≤ 6.0%). In contrast, 42% of those in the RYGB group (p = 0.002) and 37% in the SG group (p = 0.008) successfully reached these targets. These results demonstrate the superior efficacy of metabolic surgery compared to medical therapy alone. Furthermore, the positive effects of metabolic surgery were shown to be long-lasting, with even greater benefits observed at the five-year mark compared to medical treatment. This underscores the durability and sustained impact of surgical intervention in managing obesity-induced complications such as diabetes [19].

In a clinical cohort study involving 1787 veterans, the effectiveness of bariatric surgery was evaluated for patients who underwent Roux-en-Y gastric bypass (RYGB) and other forms of metabolic bariatric surgeries. The results are summarized in Table 1 [20].

**Table 1:** Effectiveness of bariatric surgery in veterans for weight loss RYBG: Roux-en-Y gastric bypass; SG: Sleeve gastrectomy; AGB: Adjustable gastric banding

Time Duration	Weight Loss Percentage-Surgical (Mean)	95% Confidence Interval
4 years	RYGB: 27.5%	23.8% to 31.2%
	AGB: 10.6%	0.6% to 20.6%
	SG: 17.8%	9.7% to 25.9%
	RYGB vs. AGB: 16.9% more in RYGB	6.2% to 27.6%
	RYGB vs. SG: 9.7% more in RYGB	0.8% to 18.6%
10 years	RYGB: 21%	11% to 31%

As evident from the aforementioned data, at the four-year mark, veterans who underwent RYGB surgery experienced significantly more weight loss compared to those who had SG or AGB.

Effectiveness and mechanism of weight loss using semaglutide

GLP-1, a hormone crucial for regulating appetite and digestion, is produced in two primary locations within the body: L-cells in the gastrointestinal tract and specialized cells in the hindbrain's nucleus tractus solitarius. The effects of native GLP-1 are mediated through GLP-1 receptors present in various tissues throughout the body. GLP-1 orchestrates a wide array of physiological actions, including enhancing the feeling of fullness, diminishing appetite, and decelerating the process of gastric emptying [21].

Semaglutide, having 94% structural homology with native human GLP-1, exhibits three important modifications. An amino acid substitution at position 8 reduces susceptibility to degradation by the dipeptidyl peptidase-4 inhibitor (DPP-4). Lysine acylation at position 26, along with a spacer and C-18 fatty di-acid chain, facilitates strong and specific binding to albumin. Additionally, an amino acid substitution at position 34 prevents unintended binding of the C-18 fatty di-acid chain. These structural modifications enhance semaglutide's stability, duration of action, and dosing convenience [11]. Semaglutide obtained approval as an anti-diabetic medication from the Food and Drug Administration (FDA) on December 5, 2017, and from the European Medicines Agency (EMA) on February 8, 2018 [22-23]. When used alongside lifestyle interventions, pharmacotherapies can be beneficial for individuals with obesity, enabling them to achieve significant weight loss and sustain it [24-25]. However, the options for pharmacotherapy have historically been limited, necessitating the development of treatments that can effectively induce and maintain clinically meaningful weight loss while also addressing complications like type 2 diabetes (T2D) and cardiovascular disease (CVD) [26-27]. Various agents for weight management have been approved or are currently under investigation. One such agent is semaglutide, a glucagon-like peptide-1 receptor agonist (GLP-1RA), initially approved for treating T2D. Semaglutide has demonstrated improvements in cardiovascular outcomes for these patients and has now received approval in 2021 for chronic weight management (2.4mg once weekly) in obese or overweight individuals [28-29]. Semaglutide exerts its effects on glucose metabolism through multiple mechanisms. It stimulates insulin secretion from beta-pancreatic cells while simultaneously reducing glucagon production from alpha-pancreatic cells, both in a glucose-dependent manner. This results in decreased fasting and postprandial plasma glucose levels [30]. Additionally, semaglutide has been shown to delay gastric emptying, which further contributes to improved glycemic control. Slowing down the rate at which the stomach empties helps prevent rapid spikes in blood glucose levels after meals [31]. In fact, semaglutide offers the additional benefit of promoting weight loss. By targeting specific receptors in the brain, semaglutide helps regulate appetite and increase feelings of satiety, leading to reduced food intake and subsequent weight reduction [32]. The Semaglutide Treatment Effect in People (STEP) with obesity trials cannot be ignored while talking about semaglutide as pharmacotherapy for weight loss. For example, the STEPs 1-5 trials, classified as phase 3, double-blind, randomized trials, aimed to assess the efficacy of once-weekly subcutaneous semaglutide 2.4 mg versus placebo in managing weight in adults with obesity or overweight [33-37]. These trials encompassed both individuals with type 2 diabetes (T2D) and those without T2D. STEPs 1-3 involved a 68-week treatment period, with an additional 45-week follow-up for participants in STEP 1, totaling 52 weeks off-treatment until the end-of-trial visit at week 120 [33]. STEP 4 incorporated an initial 20-week semaglutide run-in phase, followed by randomization to either continued semaglutide 2.4 mg or a switch to placebo for the remaining 48 weeks [36]. STEP 5 spanned a comprehensive 104-week treatment period [37]. Throughout these trials, a gradual dose escalation strategy over the initial 16 weeks was implemented to mitigate potential side effects. In addition to medication, participants also received lifestyle intervention or intensive behavioral therapy (STEP 3 only), which included 30 therapy sessions, physical activity guidance, and an initial eight-week low-calorie diet with partial meal replacement, followed by a hypocaloric diet [35].

The outcomes of the STEP 1-3 trials revealed significant weight loss of 15% to 16% in participants without T2D and 9.6% in those with T2D, achieved through once-weekly subcutaneous semaglutide 2.4 mg in adults with overweight or obesity [33-35]. In STEP 4, discontinuing semaglutide after the 20-week run-in period resulted in weight regain, whereas continuing treatment with semaglutide 2.4 mg led to further substantial weight loss after 48 weeks [36]. Encouragingly, the improvements in weight loss were sustained over the longer-term duration of 104 weeks in STEP 5 [37]. The findings from the STEP 4 and 5 trials underscore the chronic nature of obesity and underscore the significance of prolonged treatment to attain and maintain clinically meaningful weight loss. Importantly, a significantly higher proportion of patients achieved clinically meaningful weight losses of ≥5% with semaglutide 2.4 mg compared to placebo across all the STEP 1-5 trials. These results highlight the potential efficacy of once-weekly subcutaneous semaglutide 2.4 mg as a viable approach for weight management in individuals with obesity or overweight [33-37].

Multiple studies have been conducted to assess the effects of semaglutide on weight loss. Some of these are summarized in Table 2, highlighting the impact of semaglutide on body weight reduction, as noted from a review of clinical trials [38].

**Table 2:** Summary of clinical trials kg: kilograms; ER: extended release; mg: miligrams

Clinical Trial	Participants	Treatment Duration	Semaglutide dose	Key findings
Pioneer 1	703 patients	26 weeks	3, 7, or 14 mg oral semaglutide	Statistically significant reductions in body weight compared to placebo at higher doses (7 mg, 14 mg)
Pioneer 2	822 patients	52 weeks	Oral semaglutide 14 mg vs. empagliflozin 25 mg	Significant weight loss was observed with both treatments, but greater weight losses was seen with oral semaglutide at 52 weeks
Pioneer 6	3183 patients	15.9 months	Oral semaglutide 14 mg vs. placebo	Semaglutide group showed a change in body weight of -4.2 kg vs. -0.8 kg in the placebo group
Pioneer 8	729 patients	52 weeks	Oral semaglutide 3, 7, or 14 mg vs. placebo	Statistically significant reduction of >5% body weight compared to placebo over 52 weeks, dose-dependent fashion
Sustain 3	813 patients	56 weeks	Once-weekly semaglutide 1.0 mg vs. exenatide ER 2.0 mg	Mean reduction of 5.6 kg body weight with semaglutide vs. exenatide ER, higher weight loss response (≥5%) with semaglutide
Sustain 5	397 patients	30 weeks	Subcutaneous semaglutide added to basal insulin vs. placebo	Semaglutide group showed a >5% reduction in body weight in 66% of participants vs. 11% in the placebo group
Sustain 6	3297 patients	104 weeks	Once-weekly subcutaneous semaglutide 0.5 mg or 1.0 mg vs. placebo	Mean body weight in the semaglutide group was 2.9 kg lower (0.5 mg) and 4.3 kg lower (1.0 mg) than placebo
Step 1	1961 participants	68 weeks	Subcutaneous semaglutide 2.4 mg vs. placebo	Mean change in body weight was -14.9% with semaglutide vs. -2.4% with placebo, with a higher percentage of participants achieving >5%, >10%, and >15% weight reduction with semaglutide
Step 3	611 participants	68 weeks	Subcutaneous semaglutide 2.4 mg vs. placebo	Mean body weight change of -16.0% with semaglutide vs. -5.7% with placebo; additional weight reduction with semaglutide when used with dietary modifications
Step 4	803 participants	Up to 68 weeks	Continued subcutaneous semaglutide 2.4 mg vs. placebo	Participants who continued semaglutide lost an additional 10% of their body weight, with a mean change in weight of -7.9% vs. +6.9% in the semaglutide and placebo groups, respectively, from week 20 to week 68

Based on the data presented in the table, the clinical trials consistently demonstrate that semaglutide is effective in promoting weight loss across various patient populations. Semaglutide, whether administered orally or subcutaneously, consistently leads to statistically significant reductions in body weight compared to placebo or other antidiabetic agents. The weight loss achieved with semaglutide is dose-dependent, with higher doses generally resulting in greater weight reductions. Additionally, semaglutide has been shown to be effective in reducing body weight when added to insulin therapy or used in combination with other medications. These findings support the conclusion that semaglutide is an effective treatment option for weight management in patients with type 2 diabetes, obesity, or overweight individuals.

Complications of metabolic surgery vs. semaglutide for weight reduction

Metabolic surgery has remained one of the preferred choices for weight loss; however, despite having a plethora of benefits, it also has certain complications [39]. These complications can affect various systems in the body, leading to a range of symptoms and requiring careful monitoring and management post-surgery [40].

One of the most common complications of bariatric surgery is nutritional deficiency [41]. Malabsorptive surgeries, such as gastric bypass or duodenal switch, restrict the absorption of nutrients, which can result in malnutrition and deficiencies in essential vitamins and minerals [41]. Patients may experience deficiencies in iron, vitamin B12, folate, calcium, and vitamin D, leading to conditions such as anemia, osteoporosis, and neuropathy [41-43]. Regular monitoring of nutritional status and appropriate supplementation are essential to prevent and manage these deficiencies.

Hepatobiliary complications can also occur after bariatric surgery. The formation of gallstones is a common complication [44]. Gallstones may cause symptoms such as abdominal pain, nausea, and vomiting. Hence, to avoid this unwanted aftereffect, cholecystectomy is carried out in high-risk patients [44].

Dumping syndrome is another significant complication that can occur after bariatric surgery, particularly gastric bypass [41]. This syndrome is characterized by symptoms such as abdominal pain, nausea, sweating, and diarrhea, which occur after the consumption of high-sugar or high-fat meals [40]. Dumping syndrome can significantly impact a patient's quality of life and dietary choices, requiring dietary modifications and potentially medication to manage symptoms [40].

Gastrointestinal complications can also arise after bariatric surgery. Gastric ulcers may develop due to changes in stomach acid production and the use of nonsteroidal anti-inflammatory drugs (NSAIDs) for pain management [45-46]. Bowel transit issues, such as diarrhea or constipation, can occur due to changes in the anatomy and function of the gastrointestinal tract [47]. Moreover, side effects like internal and external hernias, bowel adhesions, and cases of bowel obstruction post-surgery deserve more than a casual mention. In fact, several of these patients had to undergo reoperation once these side effects became very prominent [47]. These complications may require medical intervention and dietary adjustments to alleviate symptoms and promote proper bowel function [47].

Neurological complications, although rare, can occur after bariatric surgery [48-49]. Post-RYGB, certain regions of the gut become ineffective in the physiologic absorption of some substances due to several reasons, which include but are not limited to bypassing the stomach (95%), duodenum (its alkaline secretions with high concentrations of digestive pancreatic enzymes and bile), and jejunum. Consequently, the part that receives all the medications and food is the small-volume upper gastric pouch, which finally empties into the ‘Roux limb’. This mechanism has been shown to be the culprit in the malabsorption of several psychiatric medications, manifesting as unsatisfactory mental outcomes [48]. Thiamine deficiency, specifically vitamin B1 deficiency, is a common cause of these complications. Patients who experience repetitive vomiting, often seen in conditions like persistent gastric intolerance or gastroesophageal reflux disease (GERD), are at a higher risk. Neurological complications can manifest as neuropathy, myopathy, and encephalopathy [49]. Late-onset Wernicke's encephalopathy, characterized by the triad of inattention, ataxia, and ophthalmoplegia, is a medical emergency that requires immediate treatment with parenteral thiamine. Peripheral neuropathy, presenting as mononeuropathy multiplex, can also occur [48-49].

Gynecological complications are also worth considering. Obesity itself can cause infertility due to hormonal imbalances [50]. After bariatric surgery, many obese women may no longer rely on contraception, but it is important to note that oral contraception is not effective after malabsorptive surgeries. Patients are advised to avoid pregnancy within two years after surgery, as this period is associated with maximum weight loss and potential nutritional deficiencies [50]. Vitamin profiles, including folate and vitamin B1 if vomiting is common, should be assessed before pregnancy to ensure a healthy pregnancy and proper development of the child [50].

In conclusion, bariatric surgery can lead to a range of complications affecting different systems in the body. Nutritional deficiencies, hepatobiliary complications, dumping syndrome, gastrointestinal issues, neurological complications, and gynecological complications are among the potential risks. Close monitoring, regular follow-up appointments, and appropriate management strategies are necessary to detect and address these complications effectively, ensuring the best possible outcomes for patients undergoing bariatric surgery.

On the other hand, there are patients who tend to lean towards noninvasive techniques such as pharmacotherapy for weight loss, and semaglutide has proven to be markedly successful in this domain; however, like most of the other therapeutic drugs, this comes with a sideline of adverse effects. The clinical trials mentioned above for semaglutide also precipitated certain side effects, some of which are summarized in Table 3 [33-37].

**Table 3:** Side effects of semaglutide as noted in some clinical trials STEP: Semaglutide Treatment Effect in People With Obesity

Study	Adverse effects	Incidence
Pioneer 1	Nausea and vomiting	5.1% to 16%
Pioneer 2	Nausea, diabetic retinopathy, malignant neoplasms	19.8 %, 3.4%, 1.7%, respectively
Pioneer 8	Nasopharyngitis	9.9% to 14.7%
Sustain trials	Gastrointestinal adverse effects, diabetic retinopathy, acute pancreatitis, gallbladder-related adverse events, neoplasms	38.5% to 52.3%, 1.0%, 1.0%, 2.0%-7.0%, 1.0%-3.0%, respectively
STEP 1	Upper respiratory tract infection, cholelithiasis, malignant neoplasms	8.7%-12.2%, 0.6%-1.8%, 1.0%, respectively
STEP 3	Cholelithiasis, psychiatric disorders	1.0%-3.2%, 11.8%-14.7%, respectively

Based on the available information, the gastrointestinal (GI) side effects associated with semaglutide, such as nausea, diarrhea, vomiting, and constipation, are generally self-limiting. This means that they typically resolve on their own without the need for permanent treatment discontinuation. Most of these GI side effects were reported to be mild-to-moderate in severity and transient in nature.

In the STEP and sustain trials, there have been reports of thyroid cancer cases in participants treated with semaglutide. However, it is important to note that the incidence of thyroid cancer in these trials was relatively low and overall not statistically significant compared to the control groups [33-37]. In the STEP 1-5 trials, which evaluated semaglutide for obesity treatment, the incidence of thyroid cancer was 0.2% in the semaglutide group and 0.1% in the control group. In the sustained trials, which studied semaglutide for the treatment of type 2 diabetes, the incidence of thyroid cancer was 0.2% in the semaglutide group and 0.3% in the control group [34-38]. Although a potential association between semaglutide and thyroid cancer has been observed, it is important to consider the overall low incidence and the need for further research to fully understand the relationship. Regulatory authorities continue to monitor the safety of semaglutide and other similar medications.

The main finding from the STEP 1 trial extension is that after a significant reduction in body weight during 68 weeks of treatment with once-weekly subcutaneous semaglutide plus lifestyle intervention, most of the weight loss was regained within one year after treatment withdrawal. Additionally, some cardiometabolic variables showed a similar change back to baseline levels [34]. This finding highlights the importance of continued treatment with semaglutide to maintain weight loss and the associated cardiometabolic benefits. The study suggests that the benefits observed during the treatment period may not be sustained without ongoing intervention [34]. It is worth noting that this trial extension had some limitations, such as a relatively small sample size and the potential for selection bias. However, the findings provide valuable insights into the real-world effects of semaglutide withdrawal and emphasize the need for further research and longer follow-up studies to determine the long-term outcomes of treatment discontinuation.

The choice between surgery and semaglutide for weight reduction should be based on individual factors and preferences. Bariatric surgery has demonstrated high efficacy but carries a higher risk of complications, while semaglutide offers notable weight reduction benefits with a lower risk profile. Long-term considerations, patient preferences, and multidisciplinary care should be taken into account when making a decision.

While several effects and adverse effects of surgical and pharmacologic interventions for weight loss have been discussed, continuous research is required to understand the long-term outcomes of the treatment modalities. For example, the participants of the STEP trials were roughly between one and two thousand in number, which limits the trial’s ability to showcase semaglutide’s variable effects on larger populations over a longer period of time. In addition to this, the participant number for the pioneer trials remained ever lower than 1000 in instances, further perpetuating the need to have ongoing research in this field with regular follow-ups. As a result, even though research has shown promising effects of semaglutide on weight loss, further studies, including a more diverse variety of ethnicities, are required to know the precise effects of the drug before it develops this niche role for weight loss in the market.

## Conclusions

In conclusion, semaglutide shows promise as a noninvasive pharmacotherapeutic armamentarium for weight loss, demonstrating notable efficacy. However, it is important to acknowledge the potential adverse effects, such as gastrointestinal disturbances, increased pulse rate, and rare cases of thyroid cancer. While the incidence of these side effects is relatively low, ongoing research is needed to better understand their relationship with semaglutide. The long-term effects of treatment withdrawal indicate partial weight gain and the need for continued intervention. When choosing between bariatric surgery and semaglutide, individual factors and preferences should be considered. Bariatric surgery offers high efficacy but carries a greater risk of complications, while semaglutide provides significant weight reduction benefits with a lower risk profile. A comprehensive approach involving long-term considerations, patient preferences, alternative treatment modalities, and multidisciplinary care is crucial. Further research and longer follow-up studies are necessary to optimize the use of semaglutide and fully comprehend its long-term outcomes and safety profile in clinical practice. This review has gathered information from clinical trials and other forms of previously published data to provide a comparison between bariatric surgery and semaglutide for effective weight loss, which will allow readers to access updated developments in this field of medicine.
